# Brook House immigration removal centre

**DOI:** 10.1192/bjb.2023.97

**Published:** 2024-02

**Authors:** Giuseppe Spoto

**Affiliations:** FRCPsych, MA, FAcadMEd, MEWI, Consultant Psychiatrist, Independent Doctors Federation, London, UK. Email: giuseppe.spoto@ntlworld.com

The findings of the public inquiry into the immigration removal centre (IRC) known as Brook House (BBC News, 19 September 2023) are alarming but in my opinion not altogether surprising.

In 2011, I was employed to undertake a pilot study at the IRC on behalf of my then employer, Sussex Partnership NHS Foundation Trust, with a view to improving services. The study lasted a total of 6 months, and detailed proposals were made; however, my recommendations were shelved.

I am glad to hear about the suggestion made by the inquiry chair Kate Eves that the law be changed to limit the duration of detention at IRCs such as Brook House.

I have for many years as a consultant psychiatrist and expert witness in immigration cases recommended that where the authorities are unable to make a decision about the future of a claimant in a timely fashion, the claimant should be released immediately.

It appears from the inquiry that services at Brook House are substandard at every level and that better services are needed in this critical area of practice. Psychiatry should lead as it once did in the prison service.

